# Distribution of Caskin1 protein and phenotypic characterization of its knockout mice using a comprehensive behavioral test battery

**DOI:** 10.1186/s13041-018-0407-2

**Published:** 2018-10-25

**Authors:** Tayo Katano, Keizo Takao, Manabu Abe, Maya Yamazaki, Masahiko Watanabe, Tsuyoshi Miyakawa, Kenji Sakimura, Seiji Ito

**Affiliations:** 1 Department of Medical Chemistry, Kansai Medical University, Hirakata, 573-1010 Japan; 2 Section of Behavior Patterns, National Institute of Physiological Sciences NINS, Okazaki, Aichi 444-8585 Japan; 3 Division of Experimental Animal Resource and Development, Life Science Research Center, University of Toyama, Toyama, 930-0194 Japan; 4 Department of Cellular Neurobiology, Brain Research Institute, Niigata University, Niigata, 951-8585 Japan; 5 Department of Neurology, University of California, San Francisco, 94158 USA; 6 Department of Anatomy, Hokkaido University School of Medicine, Sapporo, 060-8638 Japan; 7 Division of Systems Medical Science, Institute for Comprehensive Medical Science, Fujita Health University, Toyoake, Aichi 470-1192 Japan

**Keywords:** Caskin1, Behavioral test battery, Caskin1-KO mouse, Central nervous system, Nociception

## Abstract

Calcium/calmodulin-dependent serine protein kinase (CASK)-interacting protein 1 (Caskin1) is a direct binding partner of the synaptic adaptor protein CASK. Because Caskin1 forms homo-multimers and binds not only CASK but also other neuronal proteins in vitro, it is anticipated to have neural functions; but its exact role in mammals remains unclear. Previously, we showed that the concentration of Caskin1 in the spinal dorsal horn increases under chronic pain. To characterize this protein, we generated Caskin1-knockout (Caskin1-KO) mice and specific anti-Caskin1 antibodies. Biochemical and immunohistochemical analyses demonstrated that Caskin1 was broadly distributed in the whole brain and spinal cord, and that it primarily localized at synapses. To elucidate the neural function of Caskin1 in vivo, we subjected Caskin1-KO mice to comprehensive behavioral analysis. The mutant mice exhibited differences in gait, enhanced nociception, and anxiety-like behavior relative to their wild-type littermates. In addition, the knockouts exhibited strong freezing responses, with or without a cue tone, in contextual and cued-fear conditioning tests as well as low memory retention in the Barnes Maze test. Taken together, these results suggest that Caskin1 contributes to a wide spectrum of behavioral phenotypes, including gait, nociception, memory, and stress response, in broad regions of the central nervous system.

**Electronic supplementary material:**

The online version of this article (10.1186/s13041-018-0407-2) contains supplementary material, which is available to authorized users.

## Introduction

Calcium/calmodulin-dependent serine protein kinase (CASK)-interacting protein1 (Caskin1) was originally identified as a protein that binds to CASK [1]. Caskin1 is an adaptor protein comprising six N-terminal ankyrin repeats, an SH3 domain, two SAM domains, a proline-rich region, and a C-terminal domain. A region separating the SH3 and SAM domains of Caskin1 binds the N-terminal CaMK domain of CASK [1]. The *Drosophila* homolog of Caskin interacts with leukocyte common antigen-related (Lar) protein via its SAM domains, and this complex is required for motor axon pathfinding [2]. Nevertheless, the physiological and pathological roles of Caskin1 in mammals remain unclear.

In a previous study, we performed proteomic screening to identify PSD proteins whose expression level in the spinal dorsal horn changed in a mouse model of chronic pain in a GluN2B signaling-specific manner [3]. Caskin1 is one of the proteins identified in that screen, and its upregulation was indeed attenuated in GluN2B Y1472F knock-in mutant mice under chronic pain conditions. This mutant mouse exhibits defects not only in chronic pain transmission but also in fear-related learning [4–6], raising the possibility that a downstream molecule of GluN2B, such as Caskin1, might also play a crucial role in synaptic functions, such as pain transmission or memory formation in the brain and spinal cord.

In this study, we generated a Caskin1-specific antibody to analyze the global distribution pattern of Caskin1 in the mouse. In addition, we established Caskin1-knockout (KO) mice and subjected them to a battery of behavioral tests to characterize their neurological phenotypes.

## Results

### Generation of Caskin1-knockout (KO) mice and anti-Caskin1 antibodies

**Fig. 1:**
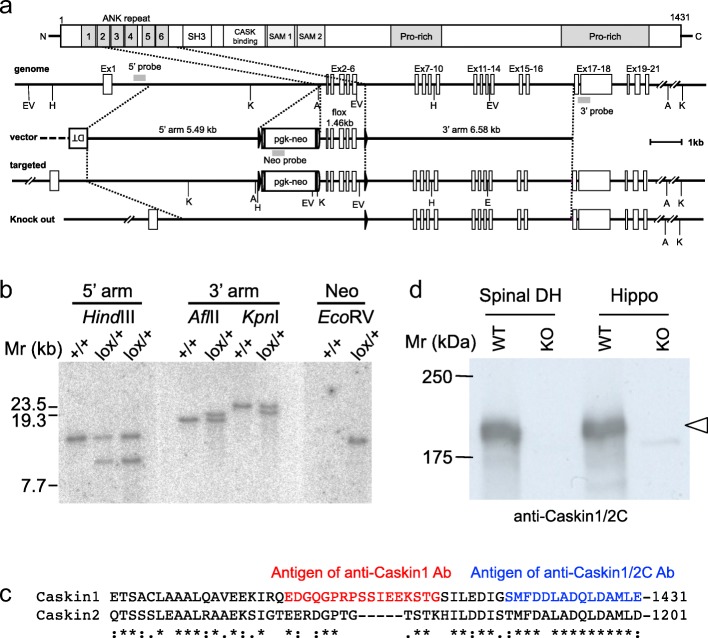
Generation of Caskin1-KO mice (**a**) Knockout strategy for the *Caskin1* gene. Homologous recombination of the targeting plasmid resulted in insertion of the *pgk-neo* cassette (*neo*) and *loxP* sequences (filled triangles) into introns 1 and 6 of *Caskin1*. Floxed mice obtained following germline transmission of ES cells harboring the homologous recombination (targeted) were crossed with “Cre-deleter” mice. Exons 2–6 of the *caskin1* gene, together with the *neo* cassette, were deleted from floxed mouse, causing a translational frameshift. A: *Afl*II, EV: *Eco*RV, H: *Hind*III, K: *Kpn*I, X: *Xba*I*.* (**b**) Southern blot analysis of homologous recombination in ES cells. Genomic DNA was prepared from wild-type (+/+) and *Caskin1* lox/+ ES cells. (Left) *Hind*III-digested DNA hybridized with a 5′ probe yielded a 12.7-kb band for the wild-type and a 9.1-kb band for the floxed allele. (Middle) *Afl*II- *or Kpn*I-digested DNA hybridized with a 3’arm probe yielded, respectively, a 16.5- or 23.5-kb band for the wild type and an 18.3- or 19.8-kb band for the floxed allele. (Right) *Eco*RV*-*digested DNA hybridized with a Neo probe yielded a 11.5-kb band for the for floxed allele. (**c**) C-terminal amino acid sequences of mouse Caskin1 and Caskin2. The red and blue sequence indicate the antigen sequences used for generation of the anti-Caskin1 and anti-Caskin1/2C antibodies, respectively. Symbols: “*”, universally conserved residue that this position; “:”, strong similarity is fully conserved; “.” weak similarity is fully conserved. **d** Western blot analysis of Caskin1 expression in spinal dorsal horn (DH) and hippocampus (Hippo) in wild-type (WT) and Caskin1-KO (KO) mouse tissues. Total proteins (20 μg/lane) were resolved on a 7.5% SDS-PAGE gel, and Caskin1 was detected with anti-Caskin1/2C antibody after blotting to a PVDF membrane

To clarify the role of Caskin1 in mammals, we generated Caskin1-KO mice. The targeting vector for the *Caskin1*-floxed mouse construct was designed as shown in Fig. 1a. Homologous recombination in ES cells was confirmed by Southern blot (Fig. 1b). *Caskin1*^*flox/+*^ mice were crossed with TLCN-Cre mice [7, 8], and the *Caskin1*^−/+^ mice were further interbred to generate *Caskin1*^−/−^ mice (Caskin1-KO). Genotyping of 535 mice revealed a normal Mendelian ratio of offspring (1, 2.056: 0.978; Table 1) from the breeding of *Caskin1*^−/+^ mice.

**Table 1:** Mendelian ratio of Caskin1-KO mice

Total Number	Genotype	Number	Rate	Theoretical rate	Theoretical number	chi-square test
535	WT	145	1	1	133.75	0.1053
	Het	277	2.056	2	267.5	
	KO	113	0.978	1	133.75	

To determine the distribution and localization of Caskin1 protein in mice, we generated a specific anti-Caskin1 antibody. In a previous study, Tabuchi et al. reported the antigen sequence for generation of an anti-Caskin1 antibody and used this antibody for Western blotting and immunohistochemistry of rat cerebellum and cultured hippocampal neurons [1]. For this study, we generated two anti-Caskin1 antibodies: anti-Caskin1/2C, obtained using the same antigen sequence reported by Tabuchi et al. (blue in Fig. 1c) [1], and anti-Caskin1, obtained using the sequence shown in red in Fig. 1c. These epitopes are distinct because the C-terminal sequence used for anti-Caskin1/2C is highly conserved between Caskin1 and Caskin2, whereas the sequence used for the anti-Caskin1 antibody is not. Caskin1 is composed of 1430 and 1431 amino acids in rat and mouse, respectively. Consistent with the previous report by Tabuchi et al. [1], using our anti-Caskin1/2C antibody, Caskin1 was detected as a ~ 180-kDa protein band in the spinal dorsal horn (DH) and hippocampus (Hippo) of wild-type mice (WT in Fig. 1d). To confirm the specificity of the anti-Caskin1/2C antibody in Western blotting, we used Caskin1-KO mice as negative controls; the 180-kDa band was not detected in these mice (KO in Fig. 1d).

### Distribution analyses of Caskin1 in mouse tissues by Western blotting

**Fig. 2:**
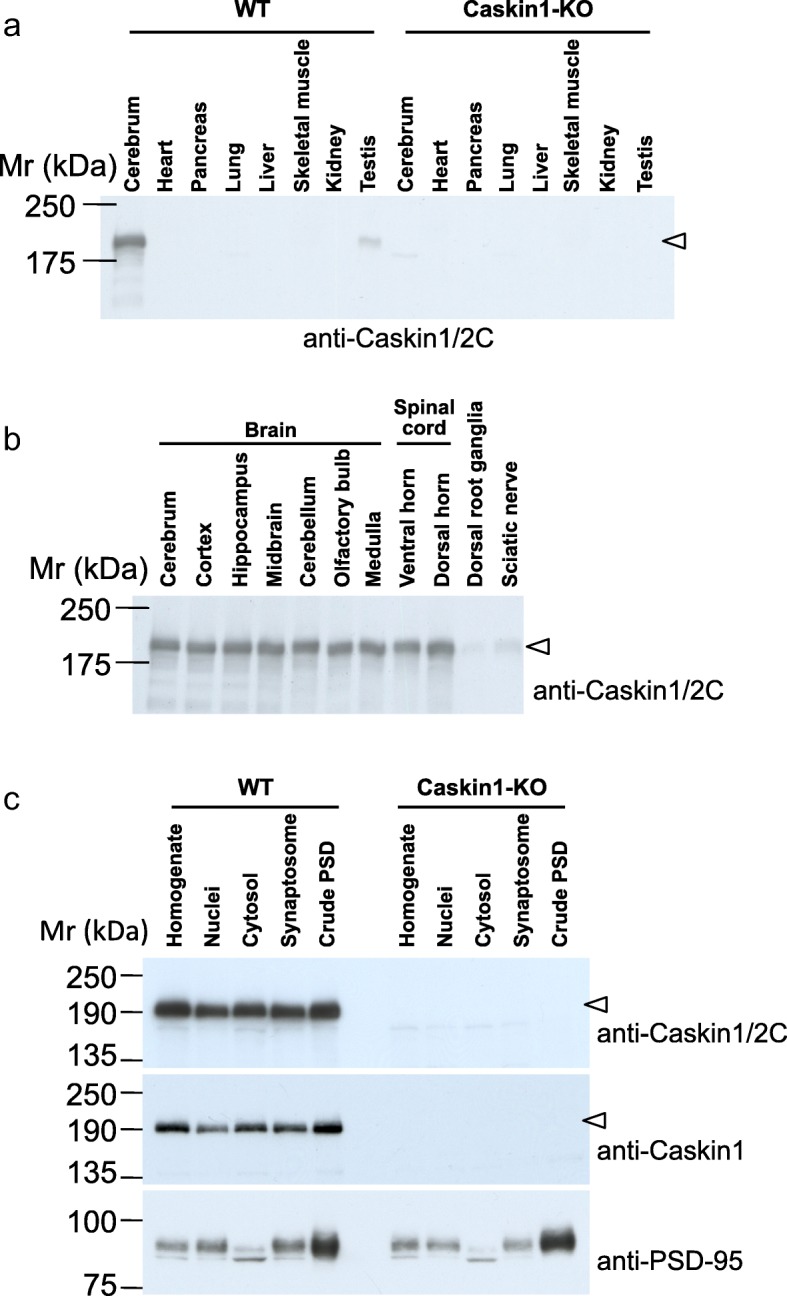
Distribution of Caskin1 in mouse tissue and specificity of anti-Caskin1 antibodies. (**a** and **b**) Western blot analysis of Caskin1 expression in wild-type (WT) and Caskin1-KO (KO) mouse tissues. Total proteins from the indicated tissues (20 μg/lane) were resolved on a 7.5% SDS-PAGE gels, and Caskin1 was detected with anti-Caskin1/2C antibody after blotting to a PVDF membrane. **c** Western blot analysis of subcellular fractions of cerebrum from WT and KO mice. Total proteins (7 μg) were reacted with anti-Caskin1/2C (top), anti-Caskin1 (middle), and anti-PSD-95 (bottom) antibodies. Arrowheads indicate the Caskin1 protein band

To clarify the tissue distribution of Caskin1 in mice, we examined homogenates from cerebrum, heart, pancreas, lung, liver, skeletal muscle, kidney, and testis by Western blotting with anti-Caskin1/2C antibody (Fig. 2a). Caskin1 was detected not only in the cerebrum, but also at low levels in testis from wild-type mice. The specificity of the anti-Caskin1/2C antibody was confirmed by the lack of reactivity in Caskin1-KO mice (Fig. 2a). In addition, we prepared several fractions from the central (cerebrum, cortex, hippocampus, midbrain, cerebellum, olfactory bulb, medulla and spinal ventral and dorsal horn) and peripheral (dorsal root ganglia, and sciatic nerve) nervous systems and subjected them to Western blotting. Caskin1 was broadly expressed at nearly equal levels in all regions of the central nervous system, but only weakly expressed in parts of the peripheral nervous system (Fig. 2b). Next, we analyzed the subcellular localization of Caskin1 by fractionation of cerebrum from wild-type and Caskin1-KO mice. Although PSD-95, a postsynaptic marker, was highly abundant in the crude PSD fraction (Fig. 2c, lower panel), this fraction was only slightly enriched in Caskin1, whereas Caskin1 was present in the other fractions (Fig. 2c upper panel). Using the Caskin1-specific antibody, Caskin1 was also detected in a pattern similar to that obtained with anti-Caskin1/2C (Fig. 2c, upper and middle panels). Neither antibody detected any Caskin1 in Western blots of samples from Caskin1-KO mice.

### Analysis of distribution of Caskin1 in the mouse brain and spinal cord by immunohistochemistry

**Fig. 3:**
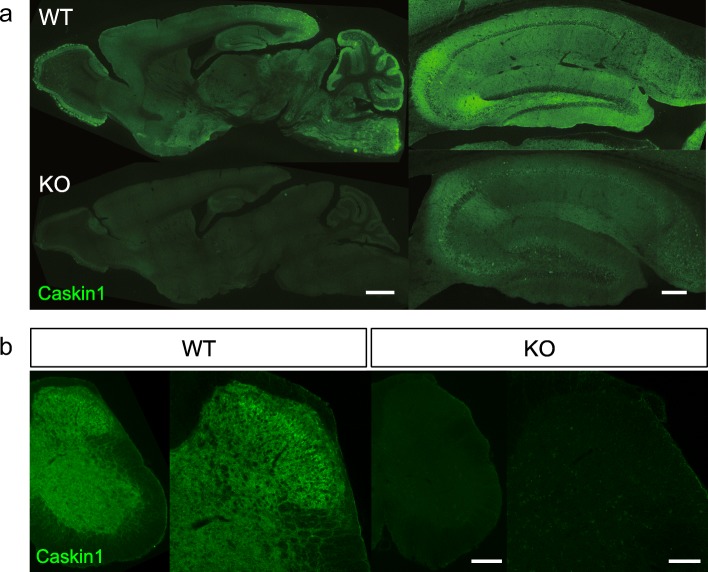
Immunohistochemistry of Caskin1 in the brain and spinal cord. **a** Sagittal sections (30 μm) of brain from wild-type (WT) and Caskin1-KO (KO) mice were prepared. Immunohistochemistry of Caskin1 in the brain was conducted using specific anti-Caskin1 antibody. Scale bars, 1000 μm (left) and 200 μm (right). **b** Transverse sections (30 μm) of spinal cord from wild-type (WT) and Caskin1-KO (KO) mice were prepared. Immunohistochemistry of Caskin1 in the spinal cord was conducted using specific anti-Caskin1 antibody. Scale bars, 200 μm (left) and 100 μm (right)

**Fig. 4:**
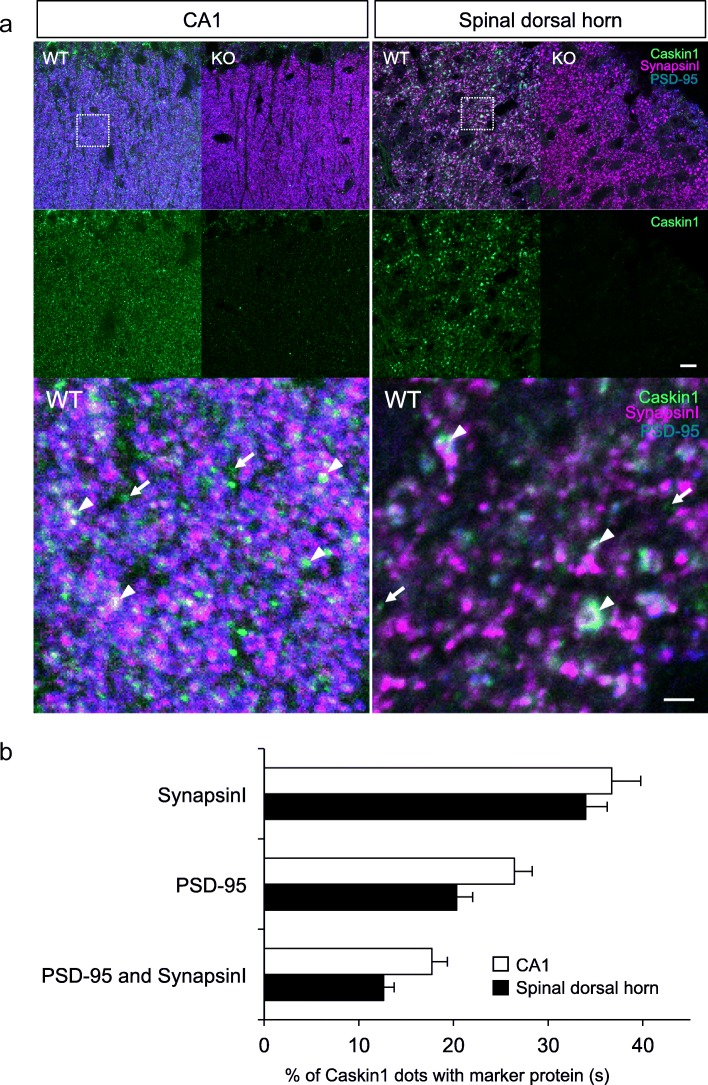
Localization of Caskin1 in the hippocampus and spinal dorsal horn. **a** Brain and spinal cord sections (30 μm) from wild-type (WT) and Caskin1-KO (KO) were prepared. Triple-staining was performed with anti-Caskin1 (green), anti-synapsin I (magenta) and anti-PSD-95 (blue) antibodies. Arrowheads indicate triple-positive dots of Caskin1, synapsin I and PSD-95, and arrows indicate Caskin1 single positive dots. Regions of high magnification of the white box in upper right of CA1 and spinal dorsal horn are shown in lower, respectively. Scale bars, 10 (upper) and 2 μm (lower). **b** Percentage of Caskin1 dots with synapse markers (synapsin I and/or PSD-95). Immunofluorescent signals were counted and calculated by use of Imaris software. All values are presented as the mean ± SEM

To determine where Caskin1 is expressed in the mouse central nervous systems, we performed immunohistochemistry with the antibodies generated for this study. Both antibodies recognized Caskin1 in brain and the spinal cord sections (Fig. 3a, b and Additional file 1: Figure S1). By contrast, the signals from both anti-Caskin1 antibodies were significantly reduced in Caskin1-KO mouse brains and spinal cord (Fig. 3a, b), although clear signals in the hippocampal somata could still be obtained with the anti-Caskin1/2C antibody (Additional file 1: Figure S1). Accordingly, we used only the Caskin1-specific antibody for immunohistochemistry. Caskin1 was previously identified as a chronic pain–related protein in PSD fractions of spinal dorsal horn [3]. Hence, we analyzed the synaptic distribution of Caskin1 in the hippocampus and the spinal cord. Specificity of the signal obtained with anti-Caskin1 antibody was confirmed using Caskin1-KO mice as negative controls (Figs. 3a, b and 4a). Caskin1 co-localized with pre- and post-synaptic marker proteins synapsin I and PSD-95 in CA1 and the spinal dorsal horn (arrows in Fig. 4a). Consistent with the results of Western blotting (Fig. 2c), Caskin1 was detected not only in synapses (as synapsin I and PSD-95 double-positive dots), but also in other regions (arrowheads and arrows in Fig. 4a). The synaptic localization of Caskin1 spots was calculated in CA1 and spinal dorsal horn from three mice, respectively. In CA1, 36.70 ± 3.03, 26.38 ± 1.91, and 17.72 ± 1.61% of Caskin1 spots were positive for synapsin I, PSD-95, and both synaptic markers, respectively (Fig. 4b). In the spinal dorsal horn, 33.96 ± 2.26, 20.32 ± 1.67, and 12.61 ± 1.12% of Caskin1 spots were positive for synapsin I, PSD-95, and both synaptic markers, respectively (Fig. 4b). These results suggest that Caskin1 localizes at synapses and at other areas in CA1 and the spinal dorsal horn.

### General health and motor function screening of wild-type and Caskin1-KO mice

To elucidate the influence of Caskin1 deletion on physical conditions, emotions, and cognitive functions in mice, we performed a battery of behavioral tests on 22 wild-type mice and the same number of Caskin1-KO mice. The mice were obtained as a single cohort by heterozygous intercrossing, and were 15–19 weeks of age at the beginning of the test battery. Subsequently, we performed the same battery of tests using the same mice between 15 and 19 and 54–59 weeks of age.

**Fig. 5:**
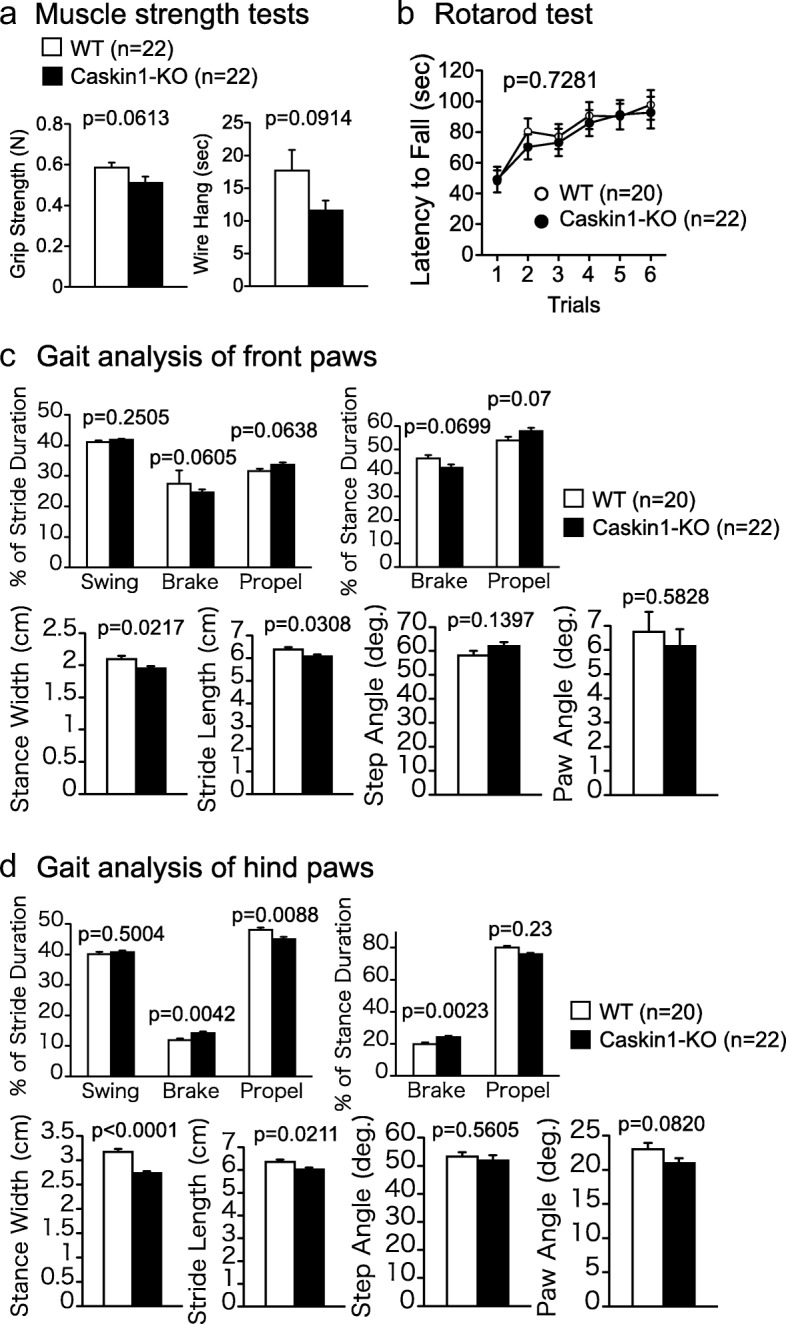
Assessment of muscle strength and motor functions in wild-type and Caskin1-KO mice. **a** Analysis of neuromuscular strength by grip strength (N) and wire hang latency (sec). *P*-values indicate genotype effect in one-way ANOVA. **b** Latency to falling off the rotating rod (sec) in the Rotarod test. *P*-value indicates genotype effect in two-way repeated-measures ANOVA. (**c** and **d**) Gait disturbance in gait analysis test of front paws (**c**) and hind paws (**d**). *P*-values indicate genotype effect in one-way ANOVA. In **c** and **d**, significance was set at α = 0.05/9 by post hoc Bonferroni’s multiple-comparison test. All values are presented as means ± SEM

First, we examined the general health and performed neurological screening (Fig. 5 and Additional file 2: Table S1). Two abnormal wild-type mice that could not open one eye were identified at this stage. The results obtained from these animals were excluded from the following behavioral analyses.

Knockout of Caskin1 caused a reduction in body weight (28.792 ± 0.681 g for KO mice vs. 32.605 ± 0.89 g for wild-type mice at 15–19 weeks of age) and body temperature (35.859 ± 0.093 °C vs. 36.327 ± 0.128 °C; Additional file 2: Table S1). In the tests for assessment of muscle strength and motor functions (grip strength, wire hang, and rotarod), no significant differences were observed between genotypes (Fig. 5a, b and Additional file 2: Table S1). These results suggest that Caskin1 is involved in regulation of body weight (i.e., balance between energy intake and energy expenditure) and some motor functions. In the gait analysis, brake (as a percentage of stride and stance duration) and stance width of hind paws differed significantly between Caskin1-KO and wild-type mice (Fig. 5d). In stride duration, brake phase was smaller and propulsion phase was greater in hind paw than in front paw, a characteristic feature of gait of rodent [9]. The proportion of the stride duration was different between front and hind paw, because the motion in gait may be also different. Thus, Caskin1-KO mice might show the significance differences only in the hind paw. In addition, smaller body-weight of Caskin1-KO mice might be related to the differences of gait.

### Abnormal anxiety-like behaviors in Caskin1-KO mice

**Fig. 6:**
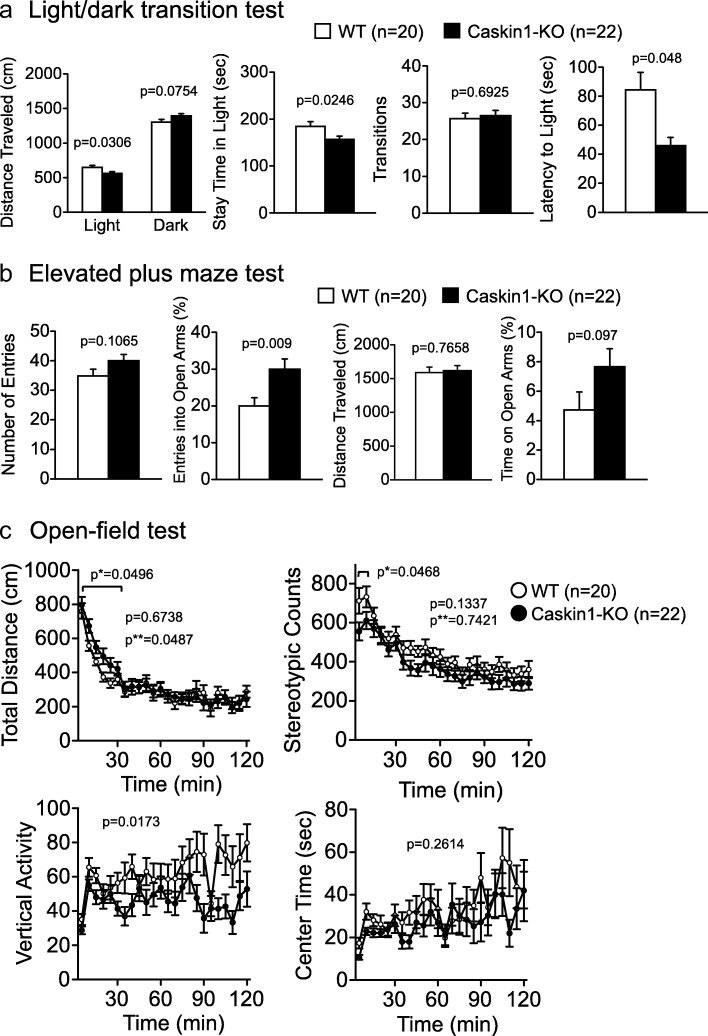
Anxiety-like behaviors tests in wild-type and Caskin1-KO mice. **a** Distance traveled (cm), stay time in light (sec), transitions, and latency to light (sec) in the light/dark transition test. **b** Number of entries, entries into open arms (%), distance traveled (cm), and time spent in open arms (%) in elevated plus maze test. *P*-values indicate genotype effect in one-way ANOVA. **c** Total distance (cm), vertical activity, stereotypic counts, and center time (sec) in the open-field test. *P*-values indicate genotype effect in two-way repeated-measures ANOVA. P* indicates significant differences in total distance in 30 min and stereotypic count in 5 min, respectively. P** indicates genotype × time interaction. All values are presented as means ± SEM

To assess anxiety-like behavior, we performed the light/dark transition, elevated plus maze, and open-field tests (Fig. 6 and Additional file 2: Table S2). In the light/dark transition test, distance traveled and time spent in the light chamber were significantly reduced in Caskin1-KO mice (Fig. 6a), suggesting elevated anxiety-like behavior. On the other hand, the first latency to enter the light chamber was significantly shorter in the knockout mice, suggesting a reduction in anxiety-like behavior. Additionally, anxiety-like behavior in the elevated plus maze test was attenuated in Caskin1-KO mice, as evidenced by their higher rate of entry into the open arms relative to wild-type mice (Fig. 6b and Additional file 2: Table S2). In the open field test, Caskin1-KO mice exhibited a significant increase in total distance within 30 min relative to wild-type mice (Fig. 6c upper left). Also, stereotypic count within 10 min and vertical activity within 120 min were significantly reduced (Fig. 6c upper right and lower left) in the mutant animals. Overall, tests of anxiety-like behavior yielded phenotypic discrepancies: the mutants exhibited a decrease in anxiety-like behavior in the elevated plus-maze test, but both an increase and decrease in such behavior in the light/dark transition and open field tests.

### Abnormal depressive-like behavior and normal prepulse inhibition in Caskin1-KO mice

**Fig. 7:**
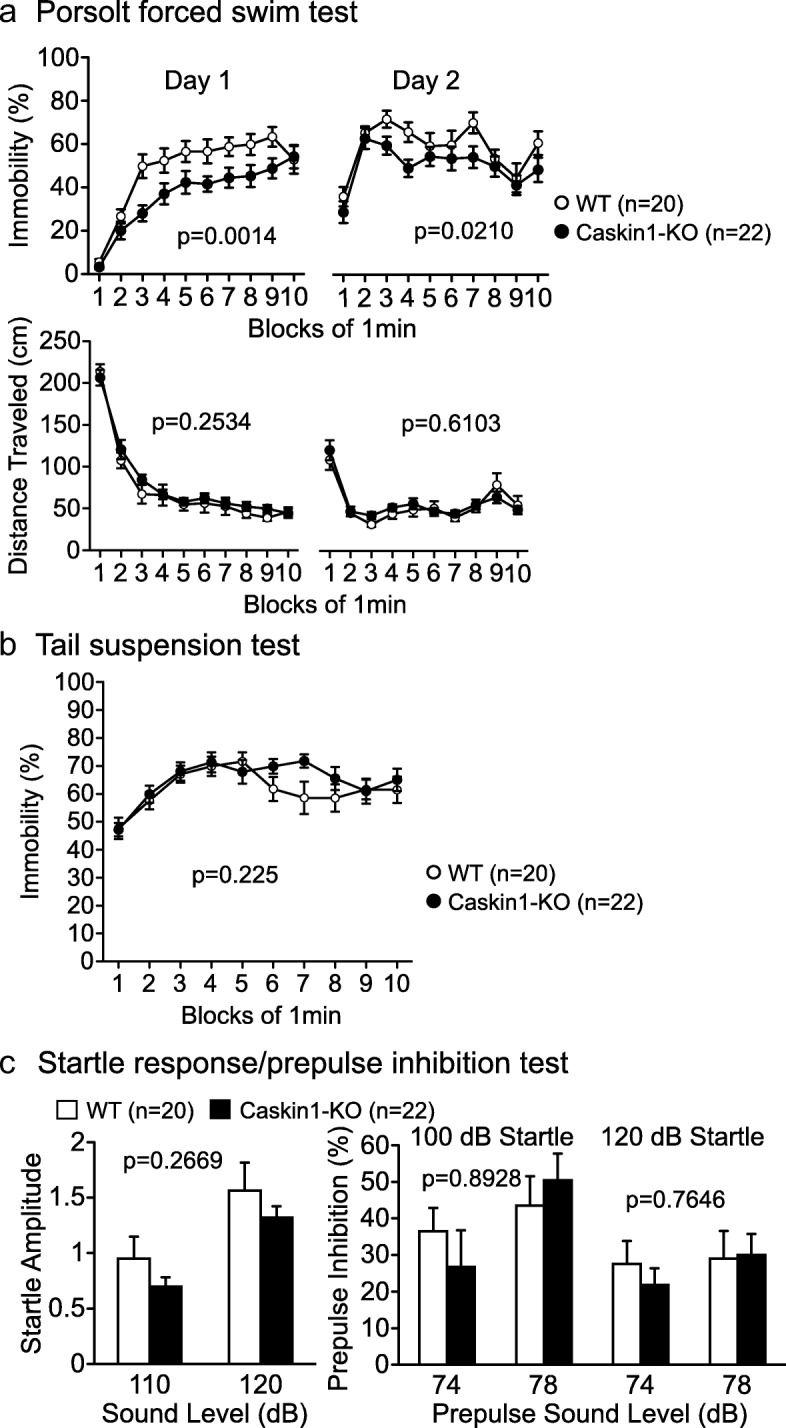
Depressive- and schizophrenia-like behaviors in wild-type and Caskin1-KO mice. **a** Immobility (%) and distance traveled (cm) on day 1 and 2 in the Porsolt forced swim test. *P*-values indicate genotype effect in two-way repeated measures ANOVA. **b** Immobility (%) in tail suspension test. *P*-values for genotype × time interaction were 0.429 (1–10 min) and 0.0578 (6–8 min). *P-*values indicate genotype effect in two-way repeated-measures ANOVA. **c** Acoustic startle response to sound stimuli (110 and 120 dB white noise) and prepulse inhibition (%) of the startle response by 74 and 78 dB prepulse stimuli in the startle response/prepulse inhibition test. *P*-values indicate genotype effect in one-way ANOVA. In C, level of significance was set at α = 0.05/6 by post hoc Bonferroni’s multiple-comparison test. All values are presented as means ± SEM

To analyze depression- and schizophrenia- related behaviors, we used the Porsolt forced swim, tail suspension, and prepulse inhibition tests (Fig. 7 and Additional file 2: Table S3). Caskin1-KO mice exhibited reduced immobility on Days 1 and 2 in the Porsolt forced swim test (Fig. 7a), indicating reduced depressive-like behavior. In the tail suspension test, immobility in the latter half of the test was modestly higher in Caskin1-KO mice than in wild-type mice, whereas genotype effect and genotype × time interactions did not differ significantly overall or during the latter half of the test (Fig. 7b). Also, we observed no significant differences in the results of the prepulse inhibition test between wild-type and Caskin1-KO mice (Fig. 7b).

### Abnormal social behaviors in home cage in Caskin1-KO mice

**Fig. 8:**
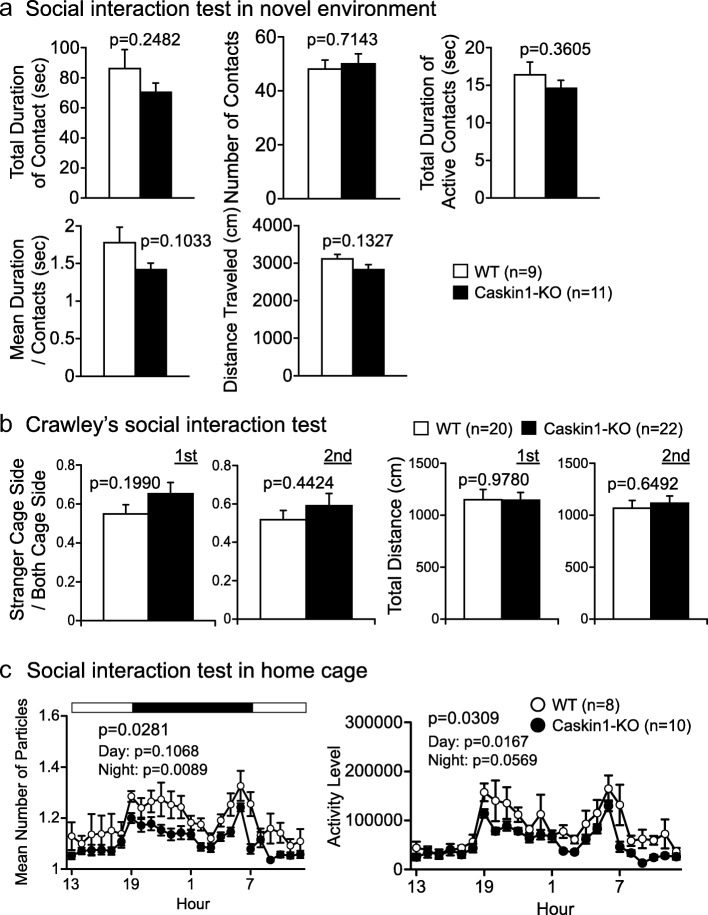
Social interaction tests in wild-type and Caskin1-KO mice. **a** Total duration of contacts (sec), number of contacts, total duration of active contacts (sec), mean duration of contact (sec), and distance traveled (cm) in the social interaction test in a novel environment. **b** Ratio of time spent on stranger cage side and total distance (cm) in Crawley’s social interaction test. P-values indicate genotype effect in one-way ANOVA. **c** Mean particle number and activity level in the social interaction test in the home cage. *P*-values indicate genotype effect in two-way repeated-measures ANOVA. All values are presented as means ± SEM

Three kinds of social interaction test (novel environment, Crawley’s version, and social interaction in the home cage) were performed to evaluate social behaviors in the Caskin1-KO mice (Fig. 8 and Additional file 2: Table S4). The novel environment and Crawley’s social interaction tests did not reveal any significant differences between wild-type and knockout mice (Fig. 8a, b). By contrast, in the social interaction test in the home cage, particle number and activity level were significantly lower in Caskin1-KO mice than in wild-type mice. Specifically, particle number did not significantly differ in the light period, but particle number during the dark period and activity level in the light period significantly lower in the mutant animals (Fig. 8c). These results suggest that sociality and basal activity in the home cage were attenuated in Caskin1-KO mice.

### Enhancement of pain sensitivity, and abnormalities in fear and spatial memory, in Caskin1-KO mice

**Fig. 9:**
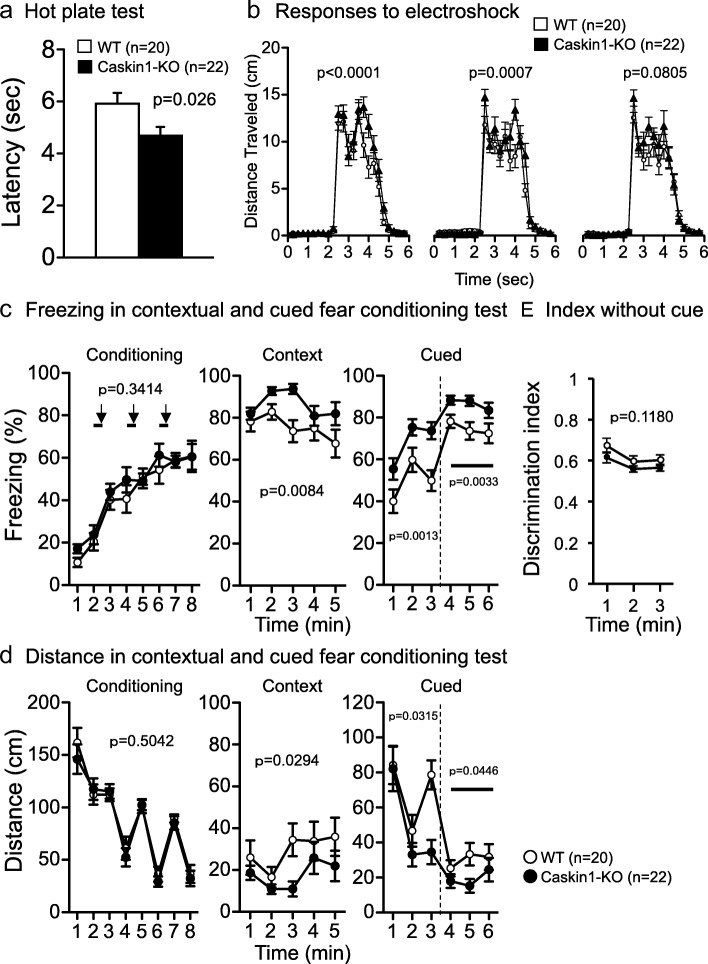
Sensitivities to noxious stimuli and contextual and cued-fear conditioning test 1 day after conditioning in wild-type and Caskin1-KO mice. **a** Latency to paw lick or foot shake (sec) on a 55 °C hot plate in the hot-plate test. (**b-d**) Context and cued-fear conditioning test. In A and B, *p*-values indicate genotype effect in one-way ANOVA. In B, level of significance was set at α = 0.05/3 by post hoc Bonferroni’s multiple-comparison test. **b** Distance traveled (cm) in 6 s with electrical stimulation (2 s) for conditioning. **c** Freezing responses on the conditioning day (left), in the contextual test 1 day after conditioning (middle), and in the cue test 1 day after conditioning (right). **d** Total distance on the conditioning day (left), in the contextual test 1 day after conditioning (middle), and in the cue test at 1 day after conditioning (right). Bold lines and arrows represent tone and footshock, respectively. **e** Discrimination index was calculated using the following formula: (a)/(a + b), where “a” equals freezing (%) at 1–3 min in the context test and “b” equals freezing (%) at 1–3 min in the cued test with altered context. In C–E, the *p*-values indicate genotype effect in two-way repeated measures ANOVA. All values are presented as means ± SEM

**Fig. 10:**
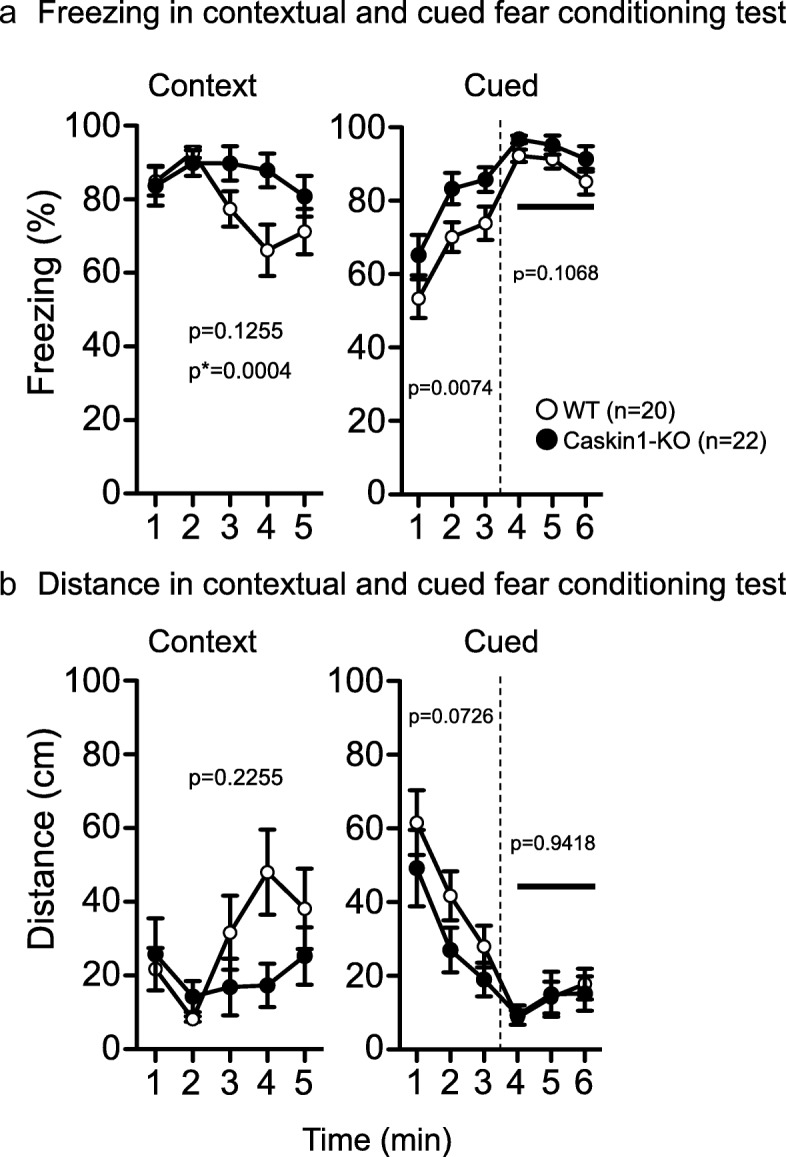
Contextual and cued-fear conditioning test at 1 month after conditioning. **a** Freezing responses 1 month after conditioning (left) and in the contextual test 1 month after conditioning (right). **b** Total distance 1 month after conditioning (left) and in the contextual test 1 month after conditioning (right). Bold lines represent the cue tone. *P*-values indicate genotype effect in two-way repeated-measures ANOVA. P* indicates genotype × time interaction. All values are presented as means ± SEM

To clarify the involvement of Caskin1 in fear memory, we performed the contextual and cued fear conditioning tests. Fear conditioning can be influenced by sensitivity to a noxious stimulus. In Caskin1-KO mice, withdrawal latency from thermal stimulation in the hot-plate test was significantly reduced (Fig. 9a and Additional file 2: Table S5). Also, the withdrawal threshold for mechanical stimuli in the von Frey test was lower in Caskin1-KO mice than in the wild-type mice (Additional file 3: Figure S2). In addition, distance moved in 6 s following the first and second footshock (for conditioning of fear memory formation) was significantly higher in the knockouts than in wild-type mice (Fig. 9b and Additional file 2: Table S4); however, the conditioning curve (total freezing and distance) in the conditioning session of the fear-conditioning test did not differ significantly between genotypes (left graph in Fig. 9c, d). Taken together, these findings indicate that Caskin1-KO mice were more sensitive to a noxious stimulus (Fig. 9a, b), whereas the effect of learning during conditioning was almost identical in mutant and wild-type mice (left graph in Fig. 9c, d). In the contextual test at 1 day after conditioning, Caskin1-KO mice exhibited a significant increase in freezing response and a significant decrease in distance traveled relative to the wild-type mice (Fig. 9c, middle and 9d, middle). Also, in the altered context chamber in the cued test (i.e., with the tone), the freezing response was significantly stronger in Caskin1-KO than in wild-type mice 1 day after conditioning (Fig. 9c, right and 9d, right). These differences in the freezing response were also observed without the tone cue in the altered context chamber (Fig. 9c, right and 9d, right). Hence, to exam the involvement of pattern separation, we calculated a discrimination index using the value at 1–3 min in the context testing and 1–3 min (without a cue) in the cued testing with altered context. Both genotypes of mice recognized the difference in the chamber well, and the values of the discrimination index did not significantly differ between wild-type and Caskin1-KO mice (Fig. 9e). The calculated index revealed that Caskin1-KO mice did not exhibit attenuation of pattern separation. On the other hand, enhancement of freezing responses in the Caskin1-KO mice was also observed 1 month after conditioning in the context testing, as well as in cued testing with altered context (Fig. 10). Contextual and cued fear memory was established in both mice groups, whereas sensitivity to nociceptive pain and overall freezing responses in the contextual and cued-fear conditioning test were higher in Caskin1-KO mice than in wild-type mice (Figs. 9c, d and 10). The elevated freezing responses in Caskin1-KO mice might be related to sensitivity to noxious stimulation.

**Fig. 11:**
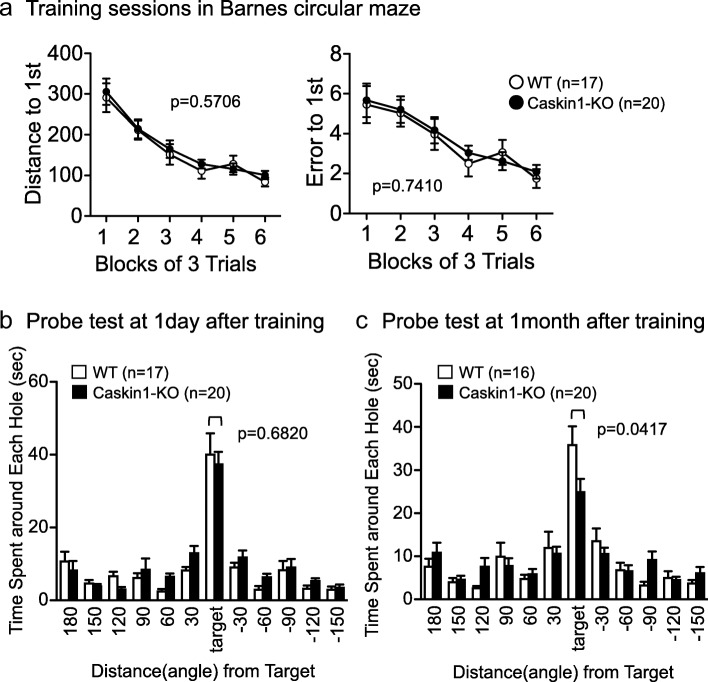
Spatial memory formation in the Barnes maze test. **a** Distance to the escape box (left) and number of errors before reaching the escape box (right). Data are presented as means of three sessions. Data were analyzed by two-way repeated-measures ANOVA. (**b** and **c**) Time spent around each hole in the probe tests conducted 1 day (**b**) and 1 month (**c**) after the last training session. *P*-values indicate genotype effect in one-way ANOVA. All values are presented as means ± SEM

To assess the involvement of Caskin1 deletion in memory independent of noxious stimuli, we performed the Barnes circular maze (Fig. 11). In 18 training sessions, the distance to the escape box and the number of errors to reach the escape box did not significantly differ between wild-type and Caskin1-KO mice (Fig. 11a). Two probe tests were performed at 1 day and 1 month after the final training sessions (Fig. 11b and c). In these tests, both wild-type and Caskin1-KO mice exhibited a significant effect of hole location (location effect: 1 day, WT *p* < 0.0001, KO *p* < 0.0001; 1 month, WT *p* < 0.0001, KO *p* < 0.0001; one-way ANOVA), indicating that both genotypes were able to distinguish the location of the target. Time spent around the correct hole did not differ significantly between genotypes at 1 day after training, but was significantly shorter in Caskin1-KO mice 1 month later (Fig. 11b and c and Additional file 2: Table S6). These results suggest that Caskin1 is involved in long-term memory.

## Discussion

In this study, we analyzed the distribution of mouse Caskin1 using new anti-Caskin1 antibodies. In addition, to assess the function of Caskin1 in vivo, we generated Caskin1-KO mice and subjected them to a behavioral test battery. Western blotting and immunohistochemical analyses using these antibodies revealed that Caskin1 was abundant in broad areas of the brain and spinal cord, but not in the peripheral nervous system (Figs. 2 and 3). Furthermore, synaptic localization of Caskin1 was confirmed in hippocampal CA1 and spinal dorsal horn (arrowheads in Fig. 4). Caskin1 signals were also detected in the other regions (arrows in Fig. 4). These results were consistent with the results of Western blotting following subcellular fractionation (Fig. 2c). Together, these findings suggest that Caskin1 localizes at both synapses and other subcellular regions.

In an earlier study, Caskin1 was identified as a PSD protein whose level increased in spinal dorsal horn neurons during chronic pain in a GluN2B signaling–specific manner [3]. This finding suggests that Caskin1 is involved in pathological pain after peripheral nerve injury in wild-type mice. Sensitivity to noxious stimuli, such as heat in the hot plate test and electroshock in the contextual and cued-fear conditioning test, was significantly higher in Caskin1-KO mice than in wild-type mice (Fig. 9). These results indicate that pain sensitivity was enhanced by Caskin1 deletion in mice without peripheral nerve injury. Therefore, it is likely that Caskin1 is closely involved in physiological and pathological pain transmission. The freezing level of Caskin1-KO mice in the contextual and cued-fear conditioning tests may be affected by their high sensitivity to nociceptive pain. In support of this idea, the higher level of freezing in Caskin1-KO mice vs. wild-type mice was independent of cue tone in cued testing with altered context (Figs. 9c, d and 10). On the other hand, retention of spatial memory 1 month after conditioning was attenuated in Caskin1-KO mice relative to wild-type mice (Fig. 11c). Spatial memory formation in the Barnes maze test is independent of any noxious stimuli. Therefore, we propose that Caskin1 is involved in both nociception and long-term memory.

In tests of anxiety-like behavior, Caskin1-KO mice exhibited phenotypic discrepancies. To explain this observation, we speculated that Caskin1-KO mice were exhibiting panic-like escape behavior due to intense anxiety. In the elevated plus maze test, Caskin1-KO mice exhibited a decrease in anxiety-like behavior, whereas in the light/dark transition and the open field tests, they exhibited both decreases and increases in anxiety-like behaviors (Fig. 6). In novel or stressful environments, mice try to escape and exhibit hyperactivity, which is interpreted as evidence of intense anxiety or a panic-like state. Homes et al. reported phenotypic discrepancies, including increased number of total, open arm, and closed arm entries in the elevated plus maze test. In addition, other anxiety-related parameters, such as head dipping also increased. Based on these results, they concluded that these phenotypic discrepancies were caused by an intense anxiety (panic-like state) [10]. Furthermore, behavioral discrepancies were reported in calcineurin-KO, Cdkl5-/Y and PSD95-KI mice in anxiety-like behavioral tests, such as light/dark transition, open field, and the elevated plus maze test [11–13]. Those reports explained and discussed their findings in terms of panic-like behavior related to intense anxiety. In the depression-like behavior test, Caskin1-KO mice also exhibited a significant decrease in immobility in the Porsolt forced swim test, as well as a non-significant increase in immobility in the tail suspension test (Fig. 7a). Similar behavioral discrepancies were also reported in Cdkl5-/Y and Sema3F-KO mice, which were explained as panic-like escaping behavior [11, 14]. Generally, mice exhibited lateral activity, an appropriate escape behavior, but those studies of Cdkl5-/Y and Sema3F-KO mice, and the result obtained with Caskin1-KO mice in this study, did not show these activities in term of distance traveled (Fig. 6a lower) [11, 14]. On the other hand, these abnormal mice exhibited a trend toward hyperactivity indicating escape motivation in one or more behavioral tests (open field, light/dark transition, elevated plus maze, Porsolt forced swim, or social interaction test) [10–15]. Moreover, hyperactivity in Caskin1-KO mice was demonstrated by numerous indices of locomotor activity, e.g., shorter latency to light in the light/dark transition test and an increased number of entries into the open arm in the elevated plus maze test. Therefore, it is likely that Caskin1-KO mice are sensitive to novel environment. Consistent with this, the mice exhibited an increase in total distance during the initial 30 min in the open field test, but not after 30 min (Fig. 6). Additionally, Caskin1-KO mice exhibited lower activity in home cage social interaction than wild-type mice (Fig. 8c). These behavioral discrepancies in Caskin1-KO mice may be related to panic-like behavior in novel environment.

Interestingly, PSD-95 and the CASK complex bind to the 5-hydroxytryptamine type 2A serotonin receptor (5-HT_2A_) [16, 17], which is involved in panic disorders [18, 19], and PSD-95 mutants exhibit panic-like behavior in a behavioral test battery [12]. Similarly, Caskin1-KO mice also exhibited features of panic-like behavior (Figs. 6 and 7). Taken together with the results of this study, these reports suggest that proteins that directly or indirectly bind 5-HT receptors are involved in panic-like disorders. Furthermore, the 5-HT_1A_-KO mice spent significantly less time in the light compartment that conditioning [20]. Heisler et al. previously reported that 5-HT_2C_-KO mice exhibited a reduction in anxiety-related behaviors [21]. Another study [22] confirmed that 5-HT_3A_-KO mice displayed reduced anxiety-related behaviors, and also showed that immobility time in the forced swim test was shorter for KO mice than wild-type mice. Given that PSD and the CASK complex can interact directly or indirectly not only with 5-HT_2A_, but also with a series of synaptic 5-HT receptors, multiple disruption of serotonergic regulation in Caskin1-KO mice may be responsible for the phenotypic discrepancies observed in this study. On the other hand, emotional anxiety is related to pain transmission, and serotonin facilitates nociceptive pain [23, 24]. Therefore, the increased pain transmission and anxiety-like behavior in Caskin1-KO mice might be mediated by 5-HT receptors.

Caskin1 might be the downstream molecule of the phosphorylation of Y1472 GluN2B, because Caskin1 was increased in the PSD fraction of the spinal dorsal horn with the phosphorylation in the chronic pain condition. However, GluN2B Y1472F knockin-mice exhibit enhanced anxiety-like behavior in the elevated plus-maze test [25] and impaired fear-related memory in auditory fear conditioning [6], both of which are contrary to the phenotypes in the Caskin1-KO mice. Accordingly, disruption of GluN2B–Caskin1 signaling might not be responsible for several of the behavioral phenotypes obtained in this study. Instead, these phenotypes may be related to other interaction proteins, such as CASK, 5-HT receptor, etc.

Caskin1, in conjunction with its interaction partners, is likely to contribute to multiple neural functions. CASK, which binds Caskin1, is a critical component of multiprotein complexes in synapses [26], suggesting that Caskin1 also plays a critical role in such complexes. Caskin1 may regulate its specific neuronal functions by controlling the composition of the CASK complex. Tabuchi et al. reported that Mint1, another component of the CASK complex, competes with Caskin1 for CASK binding in vitro, raising the possibility that Caskin1 regulates a function of the CASK–Mint1 complex, e.g., transport of NMDA and EGF receptors [27, 28].

Lar, a protein tyrosine phosphatase receptor, is another candidate binding partner of Caskin1 in mammals. A *Drosophila* homolog of Caskin associates with Lar, and this interaction is crucial for motor axon guidance [2]. In mouse, signaling by Lar and its family members is required for basal dendritic arborization during neural development [29]. Given that both Caskin1 and Lar are highly expressed in the central nervous system [30], it is possible that a Caskin1–Lar complex controls axon guidance in a broad area of the brain and spinal cord during mouse development. On the other hand, Caskin1-KO mice exhibited differences in gait and a (non-significant) decrease in wire hang relative to wild-type mice (Fig. 5a, c and d). It is likely that the role of the Caskin1–Lar complex in motor axon pathfinding is involved in these motor function abnormalities. Furthermore, Caskin1 interacts with EphB1/Nck complexes and lysophosphatidic acid [31, 32] and forms a tandem SAM domain structure mediated by Caskin1 self-association in vitro [33]. These reports regarding multiple Caskin1-associated proteins and the results of our behavioral analyses suggest that Caskin1, along with various interacting molecules, participates in multiple behaviors that are abnormal in Caskin1-KO mice.

In summary, our findings demonstrate Caskin1 is expressed in broad regions of the central nervous system, and that knockdown of Caskin1 results in multiple behavioral phenotypes. These behavioral results, together with the distribution of the protein, suggest that Caskin1 contributes to multiple neuronal functions in the brain and spinal cord. To better understand the mechanisms underlying the wide spectrum of behaviors seen in Caskin1-KO mice, future studies should seek to fully characterize the molecular functions of Caskin1 in each area of the central nervous system.

## Methods

### Animals

*Caskin1*-floxed (*Caskin1*^flox/+^) mice were produced by using the embryonic stem (ES) cell line RENKA, which was derived from the C57BL/6 N strain [34]. Homologous recombinants among the ES cells were identified by Southern blot analysis. To yield heterozygous knockout (*Caskin1*^+/−^) mice, *Caskin1*^flox/+^ mice were crossed with TLCN-Cre mice, by which recombination is induced throughout the whole body [8]. C57BL/6 N mice were used as wild-type control mice for immunohistochemistry, Western blotting, and the behavioral test battery. All behavioral testing procedures were approved by the Animal Care and Use Committee of the National Institute for Physiological Sciences. Other animal experiments were approved by the Animal Experimentation Committee, Kansai Medical University and National Institutes of Natural Sciences, and carried out in accordance with the National Institutes of Health Guide for the Care and Use of Laboratory Animals (1996).

### Antibodies

Rabbit anti-Caskin1/2C and anti-Caskin1 antibodies were raised against the C-terminal peptide SMFDDLADQLDAMLE and QEDGQGPRPSSIEEKSTG of mouse Caskin1, respectively (Fig. 1c); and anti-PSD-95 antibodies from guinea pig for immunohistochemistry were generated by Dr. M. Watanabe. Commercially available antibodies against PSD-95 (Upstate Biotech., for Western blotting) and Synapsin I (Frontier Institute) were also used.

### Subcellular fractionation of brain

After anesthesia with isoflurane, the brain was isolated and homogenized in 20 mM Tris-HCl (pH 8.0) containing 0.32 M sucrose, 2 mM DTT, protease inhibitor cocktail (Nacalai Tesque), and phosphatase inhibitor (Nacalai Tesque) by using a Potter-Elvehjem homogenizer. After centrifugation of the homogenate at 800 x g for 10 min, the pellet (nuclear) and supernatant were separated. The supernatant was then centrifuged at 13,800 x g for 20 min. The resulting pellet (synaptosomes) and supernatant (Cytosolic) were separated; and the synaptosome pellet was suspended in 20 mM Tris-HCl (pH 8.0), after which an equal volume of 1% Triton X-100 in 20 mM Tris-HCl (pH 8.0) was added to it. The synaptosome fraction was treated for 15 min at 4 °C and then centrifuged for 20 min at 15,000 rpm. The pellet was used as the crude PSD fraction (crude PSD).

### Western blot analysis

All subcellular fractions were subjected to 7.5% SDS-PAGE, and proteins were transferred to a PVDF membrane. After blocking for 1 h at room temperature with 3% skimmed milk in TBS-T buffer consisting of 0.1% Triton X-100, 150 mM NaCl, and 10 mM Tris-HCl (pH 7.5), the membrane was incubated at 4 °C overnight with rabbit anti-Caskin1/2C (1 μg/ml), anti-Caskin1 (1 μg/ml) and anti-PSD-95 (1:1000) antibodies. The membrane was then washed with the TBS-T buffer and incubated for 1 h with horseradish peroxidase-conjugated goat anti-rabbit IgG (1:20,000; Zymed) or goat anti-mouse IgG (1:20,000; GE Healthcare). It was then washed 4 times with the TBS-T buffer. The immunoreactivity was detected by use of an enhanced chemiluminescence detection kit (ECL, GE Healthcare, and Chemi-Lumi One Super, Nacalai Tesque).

### Histochemistry

Animals were anesthetized by an intraperitoneal administration of sodium pentobarbital (50 mg/kg) and perfused with 4% paraformaldehyde in 0.1 M sodium phosphate (PB, pH 7.4). Following dissection, brains and spinal cords were postfixed for 4 h in the same fixative at 4 °C and then cryoprotected overnight in 30% (*w*/*v*) sucrose in PB at pH 7.4. Sagittal sections (30-μm thick) of the brain and transverse section (30-μm thick) of the spinal cord were cut on a cryostat and processed for immunohistochemistry with anti-Caskin1/2C (1 μg/ml), anti-Caskin1 (3 μg/ml), anti-Synapsin I (1 μg/ml) and anti-PSD-95 (1 μg/ml) used as primary antibodies, with incubation overnight at 4 °C or at room temperature for 2 h. For detection of PSD-95, pepsin treatment was performed for antigen retrieval at 37 °C for 2 min in 0.2 N HCl containing 1 mg/ml pepsin (DAKO) [35]. Thereafter the sections were incubated with Alexa 488-,546- and 633-conjugated donkey anti-rabbit, −guinea pig, and -goat IgG as secondary antibody (1:300, Invitrogen) for 90–120 min at room temperature. Fluorescence images were captured with a Zeiss laser scanning confocal microscope (LSM700) or Leica fluorescent imaging system microscope (DMI 6000B), and analysis of fluorescent signals was carried out by using ZEN (Zeiss) and Imaris 8.4 (Bitplane) software.

### Experimental design of behavioral analysis

A behavioral battery test was carried out with 22 wild-type and 22 Caskin1-KO male mice, which were obtained by heterozygous intercrossing breeding. Four-to-five week-old mice were housed four (two pairs of wild-type and Caskin1-KO mice) per cage in a room with a 12-h light/ dark cycle and with access to food and water ad libitum. Behavioral testing was performed between 9:00 a.m. and 6:00 p.m. The behavioral test battery included assessment of general health, neurological screening, gait analysis, light/dark transition, open field, elevated plus maze, hot plate, social interaction (test in novel environment, Crawley’s version, and test in home cage) rotarod, startle response/prepulse inhibition, tail suspension, Porsolt forced swim, contextual and cued fear conditioning and Barnes circular maze tests [36, 37]. The interval between tests was at least 1 day. After each test, all apparatuses were cleaned with super hypochlorous water and 70% ethanol to prevent a bias due to olfactory cues. Raw data of the behavioral test and the information about each mouse are open on a public database “Mouse Phenotype Database” (http://www.mouse-phenotype.org/).

### General health and neurological screen

Body weight was measured, and neuromuscular strength was assessed by using the grip strength and wire hang tests. A grip strength meter (O’Hara & Co.) was used to assess forelimb grip strength. Mice were lifted and held by their tail so that their forepaws could grasp a wire grid. The mice were then gently pulled backward by the tail until they released the grid. The peak force applied by the forelimbs of the mouse was recorded in Newtons (N). Each mouse was tested 3 times, and the largest value was used for statistical analysis. In the wire hang test, the mouse was placed on a wire mesh that was then inverted, and the latency to fall from the wire was recorded with a 60-s cut-off time.

### Rotarod test

The rotarod test, using an accelerating rotarod (UGO Basile Accelerating Rotarod), was performed on rotating drums (3 cm diameter) and measuring the time each animal was able to maintain its balance on the rod. The speed of the rotarod was accelerated from 4 to 40 rpm over a 5-min period.

### Gait analysis

The gait of the adult mouse during walk/trot locomotion at velocities was analyzed by using DigiGait Imaging System (Mouse Specifics Inc) [38]. Equivalent stride times for fore and hind paws were composed of a shorter stance and a longer swing time. This system enables mice to walk on a motorized transparent treadmill belt, and the software automatically identifies the stance and swing components of stride, and calculates stance width, stride length, step angle, and paw angle. Briefly, we placed the mice on a treadmill belt that moves at a speed of 24.7 cm/s. We collected digital video images of the underside of mice at 150 frames per second.

### Open-field test

The apparatus was a transparent square cage (42 × 42 × 30 cm; Accuscan Instruments). Each mouse was placed in the open-field apparatus and recorded for 120 min. Total distance traveled (cm), vertical activity (rearing measured by counting the number of photobeam interruptions), time spent in the central area (20 × 20 cm), and the beam-break counts for stereotyped behaviors were measured.

### Light/dark transition test

The apparatus consisted of a cage (21 × 42 × 25 cm) divided into 2 sections of equal size by a partition with a door (O’Hara & Co.). One chamber was brightly illuminated (390 lx), whereas the other chamber was dark (2 lx). Mice were placed into the dark chamber and allowed to move freely between the chambers with the door open for 10 min. The total number of transitions between chambers, time spent in each chamber (s), latency to first enter the light chamber (s), and distance traveled in each chamber (cm) were recorded automatically by ImageLD software (see Section, “Image analysis for behavioral tests”).

### Elevated plus maze test

The elevated plus maze consisted of 2 open arms (25 × 5 cm, with 3-mm-high ledges) and 2 closed arms (25 × 5 cm, with 15-cm-high transparent walls) of the same size (O’Hara & Co.). Each mouse was placed in the central square of the maze (5 × 5 cm), facing one of the closed arms, and was recorded for 10 min. The distance traveled (cm), number of total entries into arms, percentage of entries into open arms, and percentage of time spent in open arms were calculated automatically by using ImageEP software (see Section, “Image analysis for behavioral tests”).

### Porsolt forced swim test

The apparatus consisted of a plastic cylinder (20 × 10 cm; O’Hara & Co.) placed in the center of an opaque plastic chamber (31 × 41 × 41 cm). The cylinders were filled with water (approximately 23 °C) to a height of 7.5 cm. Mice were placed into the cylinders, and the immobility and distance traveled were recorded over a 10-min test period. Retention test trials were administered 24 h after the first trials. Image data acquisition and analysis were performed automatically by using ImagePS software (see Section, “Image analysis for behavioral tests”).

### Tail suspension test

Each mouse was suspended 30 cm above the floor by the tail in a white plastic chamber (31 × 41 × 41 cm; O’Hara & Co.). The behavior was recorded for 10 min. Immobility was judged by use of ImageTS software (see Section, “Image analysis for behavioral tests”) according to a certain threshold. Immobility lasting for less than a 2 s was not included in the analysis.

### Startle response/prepulse inhibition test

A startle reflex measurement system (O’Hara & Co.) was used to measure startle response to a loud noise and prepulse inhibition of the startle response. A test session began by placing a mouse in a plastic cylinder where it was left undisturbed for 10 min. White noise (40 ms) was used as the startle stimulus for all trial types. The startle response was recorded for 400 ms starting with the onset of the startle stimulus. The background noise level was 70 dB. The peak startle amplitude was used as a dependent variable. A test session consisted of 6 trial types (e.g., 2 types of startle-stimulus-only trials, and 4 types for prepulse inhibition trials). The intensity of the startle stimulus was either 110 or 120 dB. The prepulse sound was presented 100 ms before the onset of the startle stimulus, and its intensity was 74 or 78 dB (20 ms). Four combinations of prepulse and startle stimuli were used (74–110, 78–110, 74–120, and 78–120 dB). Six blocks of the 6 trial types were presented in a pseudorandom order such that each trial type was presented once within a block. The average inter-trial interval was 15 s (range: 10–20 s).

### Social interaction test in a novel environment

Two mice of the same genotype, and that had been previously housed in different cages, were placed in a box together (40 × 40 × 30 cm; O’Hara & Co.) and were allowed to explore freely for 10 min. Mouse behavior was analyzed automatically by using ImageSI software (see Section, “Image analysis for behavioral tests”). The total duration of contacts (s), number of contacts, total duration of active contacts (s), mean duration per contact, and total distance traveled (cm) were measured. The active contact was defined as follows: images were captured at 3 frames per second, and the distance traveled between 2 successive frames was calculated for each mouse. If the 2 mice contacted each other and the distance traveled by either mouse was 5 cm and more, the behavior was considered an “active contact.”

### Crawley’s social interaction test

The Crawley’s social interaction test using three chambers, a sociability and preference for social novelty test, was performed as previously described [39, 40]. The apparatus comprised a three-chambered box (O’Hara & Co.). Each chamber was 20 cm × 40 cm × 22 cm and the dividing walls had small openings (5 cm × 3 cm) to allow exploration into each chamber. Data acquisition and analysis were performed automatically using ImageCSI (see “Image analysis for behavioral tests”). The day before testing, the mice were individually placed in the middle chamber and allowed to freely explore the entire apparatus for 10 min. In the sociability test, a stranger mouse (C57BL/6 J male) that had no previous contact with the test mice was placed in a wire cage in one of the side chambers. The placement of the stranger mouse in the left or right side chambers was systematically alternated between trials. The test mouse was first placed in the middle chamber and allowed to explore the three chambers for 10 min. The amount of time spent in each chamber and time spent around each cage were recorded automatically for 10 min for the sociability test. After the sociability test, each mouse performed the social novelty preference test for a further 10 min using a novel stranger mouse. The stranger mouse used for the sociability test stayed in the same wire cage (this mouse was considered as the familiar mouse) and a novel stranger mouse was placed in another cage on the opposite side of the chamber. The time spent in each chamber and the time spent around each cage were also recorded automatically for a second 10-min session for the social novelty preference test.

### Social interaction test in home cage

A social interaction test in a home cage was conducted as previously described [41]. Two mice of the same genotype, which had been housed separately, were placed together in a home cage. Their social interaction and locomotor activity were monitored for 7 days. Social interaction was measured by counting the number of particles detected in each frame, i.e., two particles indicated that the two mice were separate from each other, and one particle indicated that they were in contact with each other. Data acquisition and analysis were performed automatically using ImageHCSI (see Section, “Data analysis for behavioral tests”).

### Hot-plate test

Mice were placed on a 55.0 ± 0.3 °C hot plate (Columbus Instruments), and latency to the first paw response (s) was recorded with a 15- s cut-off time. The paw response was defined as either a foot shake or a paw lick.

### Von Frey test

Mechanical threshold was assessed by use of the von Frey test. Each test was started from 0.4 g as intermediate value. The filaments were inserted through the mesh floor of the cage and applied for 5 s to the plantar surface of the hind paw. The threshold was taken as the individual force required for a withdrawal reflex of the paw. Following Chaplan’s up-down methods, the threshold was confirmed showing the positive and negative responses by using upper and lower filaments, respectively [42]. The cut-off value set at 2 g [43, 44].

### Contextual and cued fear conditioning test

Each mouse was placed in a transparent acrylic chamber (33 × 25 × 28 cm) with a stainless-steel grid floor (0.2 cm-diameter, spaced 0.5 cm apart; O’Hara & Co.) and was allowed to explore freely for 2 min. Subsequently, a 55-dB white noise, which served as the conditioning stimulus (CS), was presented for 30 s. During the last 2 s of CS presentation, a mild footshock (0.3 mA, 2 s), which served as the unconditioning stimulus (US), was presented. Two more CS-US pairings were presented with a 2-min inter-stimulus interval. Twenty-four hours after the conditioning, a context test was conducted in the same chamber. A cued test with altered context was then performed in a triangular chamber (33 × 29 × 32 cm) made of white opaque plastic, which was located in a different room. In each test, freezing percentage and distance traveled were calculated automatically by using ImageFZ software (see Section, “Image analysis for behavioral tests”).

### Barnes circular maze test

The Barnes circular maze task was conducted on “dry land,” a white circular surface, 1.0 m in diameter, with 12 holes equally spaced around the perimeter (Ohara & Co., Tokyo). The circular open field was elevated 75 cm from the floor. A black Plexiglas escape box (17 × 13 × 7 cm) with paper cage bedding on the bottom was located under one of the holes. The hole above the escape box represented the target, analogous to the hidden platform in the Morris task. The location of the target was consistent for a given mouse but randomized across mice. The maze was rotated daily, with the spatial location of the target unchanged with respect to the distal visual room cues, to prevent bias based on olfactory or proximal cues within the maze. Eighteen trials were conducted on successive days as training. One day after training, a probe trial was conducted without the escape box, to confirm that this spatial task was acquired based on navigation by distal environment room cues. Another probe trial was conducted 1 month after the initial probe test to evaluate memory retention. Time spent around each hole was recorded by using the Image BM software (see Section, “Image analysis for behavioral tests”).

### Image analysis for behavioral tests

The application software used for the behavioral studies (ImageLD, EP, SI, CSI, HCSI, PS, FZ, TS and BM) were based on the public domain NIH Image program (developed at the U.S. National Institutes of Health and available at http://rsb.info.nih.gov/nih-image/) and ImageJ program (http://rsb.info.nih.gov/ij/), which were modified for each test (available through O’Hara & Co.). ImageLD [45], ImageEP [46], ImageFZ [47] and ImageBM are freely available at the following URL: http://www.mouse-phenotype.org/software.html.

### Statistical analysis

Statistical analyses for behavioral studies were conducted with StatView (SAS Institute) or GraphPad Prism7 (GraphPad Software Inc.). Data were analyzed by one- way ANOVA, two-way repeated measures ANOVA, paired t-test, or Chi-square test. Values in graphs were expressed as the mean ± SEM.

## Additional files

Additional file 1: **Figure S1.** Immunohistochemistry of Caskin1 in the brain using anti-Caskin1/2C antibody. (TIF 848 kb)

Additional file 2: **Table S1.** General health and motor functions. **Table S2.** Anxiety-like behaviors. **Table S3.** Depressive- and schizophrenia-like behaviors. **Table S4.** Social behaviors. **Table S5.** Response to electroshock and threshold of thermal stimulation. **Table S6.** Time spend around target in probe tests of the Barnes maze test. (XLSX 30 kb)

Additional file 3: **Figure S2.** Withdrawal threshold to mechanical stimulation in von Frey test. Basal mechanical sensitivity was evaluated by von Frey test. Withdrawal threshold of wild-type (WT, white column) and Caskin1-KO (black column) mice were assessed in 8–15 week-old mice. Data are expressed as described in “Material and Methods” as the mean ± SEM (*n* = 17–18). *P*-value indicates genotype effect in Mann-Whitney U-test. (PDF 55 kb)

## Abbreviations

CaMKII: Calcium and Calmodulin-dependent kinase II
CASK: Calcium/calmodulin-dependent serine protein kinase
Caskin1: Cask-interacting protein 1
Lar: Leukocyte common antigen-related
PSD: Post synaptic density
