# Supplementary figures and images for: Distribution of Caskin1 protein and phenotypic characterization of its knockout mice using a comprehensive behavioral test battery
Source: Mol Brain. 2018 Oct 25;11:63. doi: 10.1186/s13041-018-0407-2 (PMC6202847; doi:10.1186/s13041-018-0407-2)

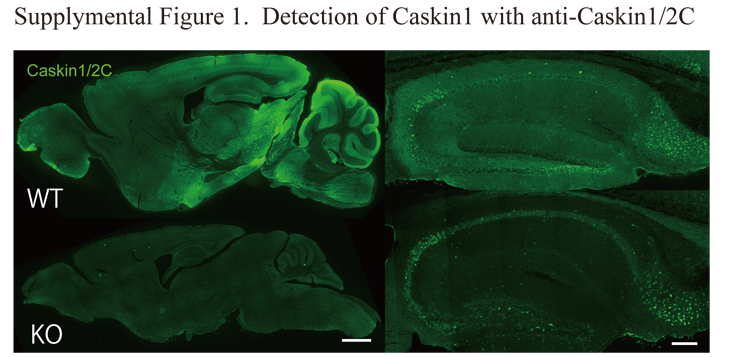

Supplement: Supplementary file 1 — Figure S1. Immunohistochemistry of Caskin1 in the brain using anti-Caskin1/2C antibody. (TIF 848 kb) [file 13041_2018_407_MOESM1_ESM.tif]

**Sup. Fig. 2 Withdrawal threshold to mechanical stimulation in von Frey test**

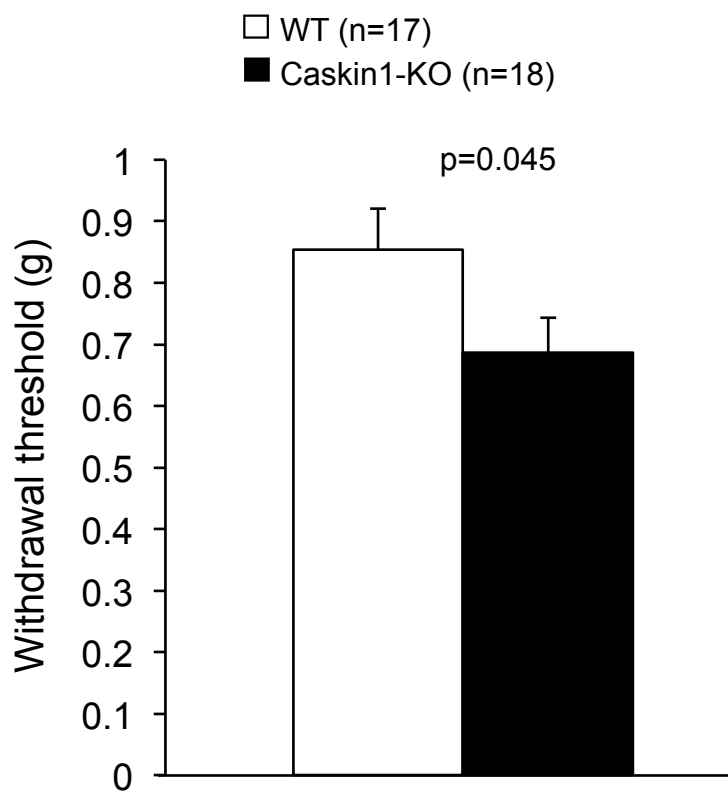

Supplement: Supplementary file 3 — Figure S2. Withdrawal threshold to mechanical stimulation in von Frey test. Basal mechanical sensitivity was evaluated by von Frey test. Withdrawal threshold of wild-type (WT, white column) and Caskin1-KO (black column) mice were assessed in 8–15 week-old mice. Data are expressed as described in “Material and Methods” as the mean ± SEM (n = 17–18). P-value indicates genotype effect in Mann-Whitney U-test. (PDF 55 kb) [file 13041_2018_407_MOESM3_ESM.pdf]
